# Supplementary figures and images for: BAZ2A-RNA mediated association with TOP2A and KDM1A represses genes implicated in prostate cancer
Source: Life Sci Alliance. 2023 Apr 25;6(7):e202301950. doi: 10.26508/lsa.202301950 (PMC10130768; doi:10.26508/lsa.202301950)

Original source data Figure 1B

Figure 1B Top panel

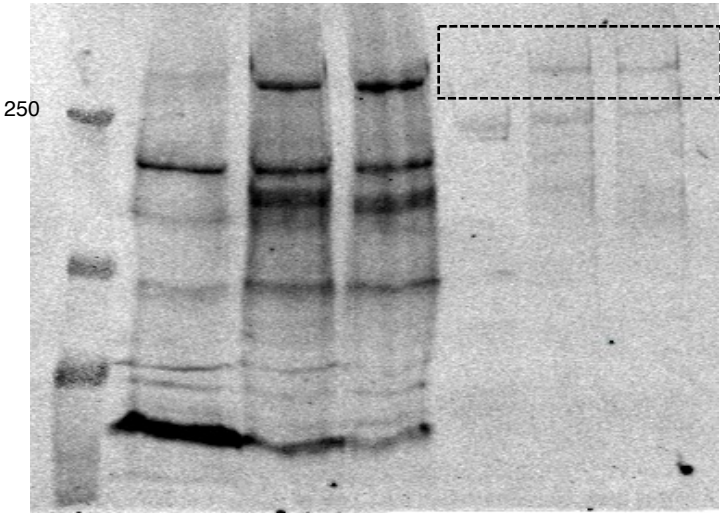

Figure 1B Bottom panel

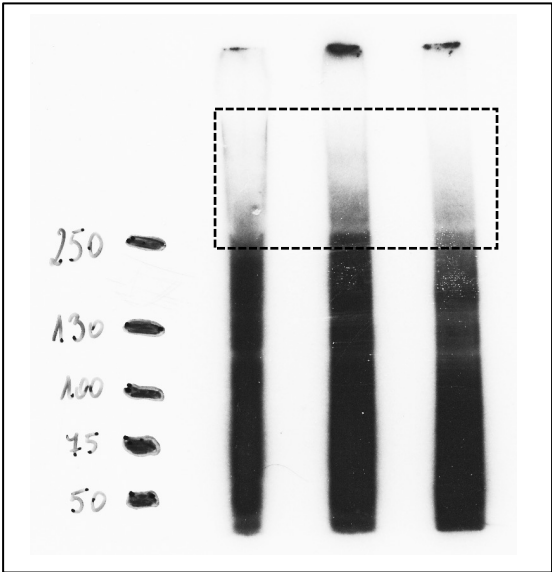

Supplement: Supplementary file 1 [file LSA-2023-01950_SdataF1.pdf]

Original source data Figure 2B

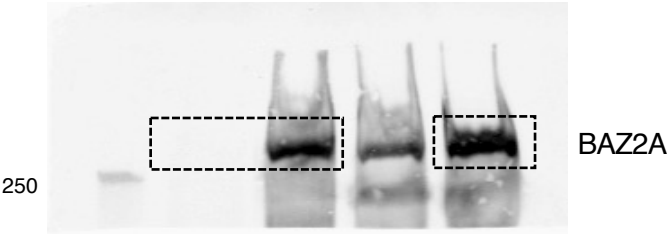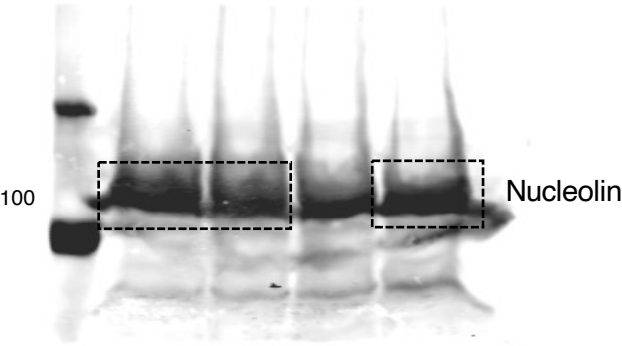

Supplement: Supplementary file 2 [file LSA-2023-01950_SdataF2.pdf]

Original source data Figure 4B

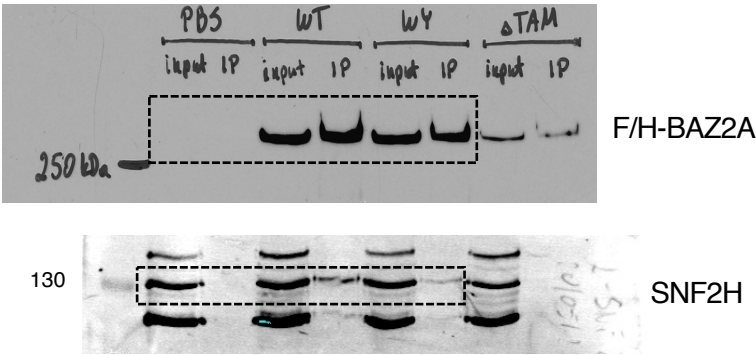

Supplement: Supplementary file 6 [file LSA-2023-01950_SdataF4.1.pdf]

Original source data Figure 4D

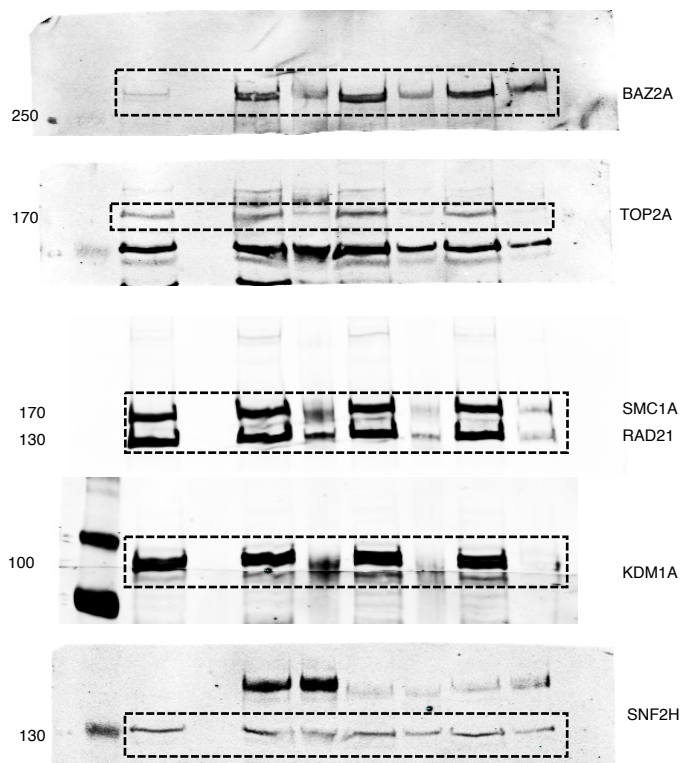

Supplement: Supplementary file 7 [file LSA-2023-01950_SdataF4.2.pdf]

Original source data Figure 4E

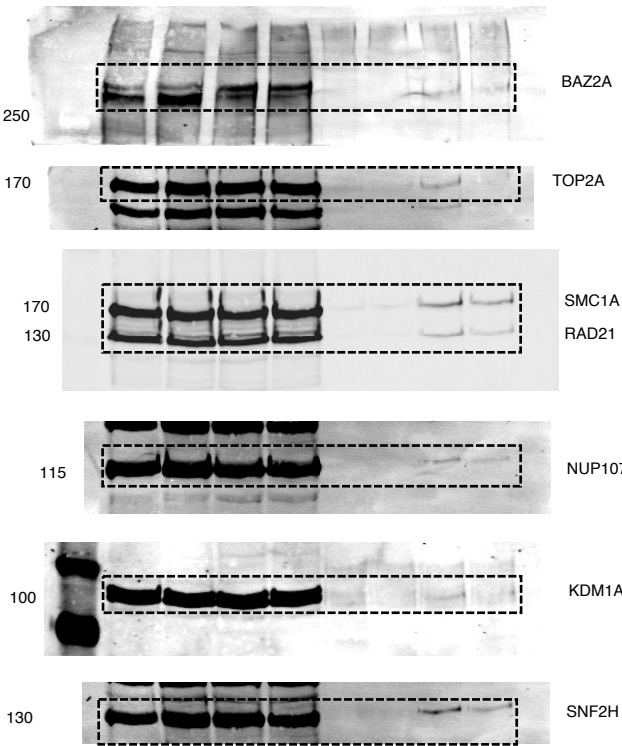

Supplement: Supplementary file 8 [file LSA-2023-01950_SdataF4.3.pdf]
